# Blue Lotus and Acute Psychiatric Symptoms: A Short Review

**DOI:** 10.1192/bjo.2026.11137

**Published:** 2026-06-30

**Authors:** Filipa M.A.A. Teixeira

**Affiliations:** South West London and St. George’s Mental Health NHS Trust, London, United Kingdom. Guy’s and St Thomas’ NHS Foundation Trust, London, United Kingdom. King’s College London, London, United Kingdom

## Abstract

**Aims:**

Legal herbal supplements are increasingly used as “natural” alternatives for mental health issues. *Nymphaea* *caerulea* (Blue Lotus) is promoted online as a relaxant, though its neuropsychiatric effects remain poorly understood. This short review aims to examine the available literature on*Nymphaea caerulea*(Blue Lotus), with a focus on its pharmacology, psychoactive and neuropsychiatric properties, and to describe illustrative examples from clinical practice. The review also seeks to raise awareness of potential associations between new-onset psychotic presentations and legal herbal supplements, and identify gaps in the evidence base requiring further research and regulation.

**Methods:**

A narrative literature search was conducted in PubMed, Google Scholar, and grey literature up to 2025 using the terms “*Nymphaea caerulea*”, “Blue Lotus”, “herbal psychoactive”, and “psychiatric symptoms”. Pharmacological sources and regulatory reports were also reviewed. Two brief anonymised clinical vignettes from clinical practice were also included to illustrate real-world psychiatric presentations temporally associated with Blue Lotus use.

**Results:**

The results of the search revealed fewer than ten publications, with only one peer-reviewed case series, which described five individuals presenting with agitation, confusion, derealisation, and anxiety following ingestion or vaping. Routine toxicology screening was negative in all cases. Two brief anonymised clinical vignettes from recent clinical practice were also included, illustrating hypomanic and psychotic presentations following Blue Lotus use.

Regulatory alerts from the UK Office for Product Safety and Standards have raised concerns around the safety of *Nymphaea caerulea* (Blue Lotus), particularly the lack of information on dosages, variability in psychoactive content, and concerns regarding purity standards. Pharmacological reports have described the main active compounds, including nuciferine and aporphine, which act on dopaminergic and serotonergic receptors and may induce both calming and euphoric effects. The concentration of these alkaloids may vary depending on the plant, extraction method, and storage, resulting in unpredictable clinical effects. Furthermore, nuciferine has been identified in Blue Lotus resin used in electronic-cigarette devices, confirming the presence of psychoactive compounds in products marketed for inhalation.

**Conclusion:**

*Nymphaea caerulea* is a legally obtainable, yet psychoactive herbal supplementthat may provoke neuropsychiatric symptoms. Clinicians should inquire about herbal/“natural” supplement use in new-onset psychiatric presentations. Regulation and further research are needed.

